# Prof. Noeggerath’s Clinic for Diseases of Females, at the N. Y. Medical College, with Remarks

**Published:** 1864-10

**Authors:** C. C. Terry


### PROF. NOEGGERATH'S CLINIC FOR DISEASES OF FEMALES, AT THE N. Y. MEDICAL COLLEGE, WITH REMARKS.

REPORTED BY C. C. TERRY, M. D. SUPERINVOLUTION—SUBINVOLUTION.

Mary P., aged 37, a strong, well developed woman has been married nine years, and has had two children ; the last is five years old, and both are healthy.

The history of this case, as derived from the patient's account indicated post-puerperal inflammation. She complained of dizziness with continual headache and flushes of heat, backache and dragging sensation in the pelvis; symptoms subject to exacerbations corresponding to monthly periods. The menses have been entirely absent during the five years succeeding the last confinement, never having appeared since the child was born, although at times the symptoms have been severe. The uterus was found very small, scarcely larger than the uterus of twelve years, and the os entirely closed, though not obliterated. The cervix was nipple-shaped, and the whole organ very movable.

Jane S., aged 25; married four years; has had two miscariages—one in the fifth month, the other in the seventh. The last miscarriage occurred eight months ago. Her habits for most of her life since the menses appeared have been sedentary; constipation has been a continual, and scanty urination an occasional inconvenience for half a dozen years. She complains of backache, especially in the lumbar region; burning in her eyes; neuralgic pains about the chest, especially under the mammæ; and loss of memory. There is constant pain in the left iliac fossa, increased by pressure, which has existed with more or less severity since the last miscarriage. The menses are regular, lasting four days, with little more than the usual pain. The uterus large and soft; the cavity enlarged and directed to the right.

B C., aged 35; was confined with her last child a year ago. Since that time she has not menstruated regularly, but has been subject to hemorrhages from the uterus at irregular periods, oftener than once a month, and at times considerable. There is pain in the right iliac region, backache especially in the lumbar region, and flour albus. The last labor was difficult; the placenta was prævia, and the child was turned. The "getting up" was tedious, lasting several weeks. Examination by the vagina revealed a large, soft tumor behind the posterior cul-de-sac, nearly obliterating it. The uterine tissue was softened; the cervix broad, but not long; the os patulous. The sound showed an increased uterine cavity and a considerable uterine retroflection, with slight lateral displacement to the right. Another case of subinvolution, complicated with anteversion, appeared once at the clinic, but it is only mentioned in these reports because the history was not sufficiently obtained.

Superinvolution and subinvolution are both results of a purely physiological process. The enlargement of uterus during pregnancy is due partly to the increased size of the muscular fibres already existing, and partly to the development and growth of new muscular fibres of precisely the same kind, and with the same tendency to increase in size as the primative fibres. After the contents of the uterus have been expelled, the need of such enlargement and muscularity is relieved, the organ atrophied; and these two wonderful changes in the condition of the uterus—the great increase before, and nearly equal decrease after paturition—are wonderful only from their rapidity; for every organ of the body, after having fulfilled its purpose is subject to the same change, however differently or slowly it may be effected. The change in the muscular fibres of the uterus, by which it returns to its unimpregnated size, is effected partly by a withering, on account of the diminished supply of blood, but mostly by fatty degeneration and subsequent absorption of the muscular fibres. These same changes of increase and decrease follow in different degrees any expansion and subsequent evacuation of the uterine cavity. It would be an interesting inquiry to know whether this degeneration affects equally the primative and the new fibres, or whether the new fibres are not reduced by mere shrinking. This process of subsidence is called involution. The uterus never recovers the same form and size it had before pregnancy.

In the virgin and nulliparious uterus, the cavity of the body is divided into two parts—one commencing at the neck, narrow and long, the other between the openings of the Fallopian tubes, formed, as it were, by two trigons connected at the bases. Thus the three sides of the cavity are convex, while the cavity of the neck is only a trifle larger than at birth, and still as long as the cavity of the body, enlarged in the middle, and nearly closed at both extremeties. The free edges of the folds representing the branches of the arbor-vitæ look downward, and may be so projected as to catch the point of the probe. The os externum is more transverse than circular. In the multiparious uterus, in its normal condition, the cavity of the body is quite triangular, still inclosed in convex lines, but much less so than in the virgin uterus. The vertical and lateral diameters of the organ are both increased; the cavity of the neck is larger, but shortened; the arbor-vitæ nearly or quite obliterated; and the os externum expanded transversely. There is thus something like a standard for the uterus when it returns after parturition, when it *involves*. If the process is carried *beyond* the standard, it is called superinvolution; if it stops *short* of the standard, it is called *sub-involution*.

The uterine atrophy, which results from excessive resorptive power is comparatively rare, but when met, is as easily diagnosed as any of the other organic diseases of the uterus. In the mere history taken from the lips of the patient, there is nothing pathognomonic of the spedial condition.—The symptoms all point to the uterus, but the physical examination is the combining and conclusive means of diagnosis.

The post-puerperal inflammation in the fourth case might as well have resulted, and usually does result, in sub-, rather than superinvolutions; the intense congestion prevents, in a great degree, the process of absorption, at the same time that the inflamatory exudation infiltrates the tissue, and contributes to its size. The total absence of the menses would suggest a much diminished secreting surface; and the sterility observed in such cases as have been reported, both of superinvolution and congenital smallness of the uterus, would suggest incapacity from smallness, since the subinvolved uterus so often conceives to expel the product of conception prematurely and repeatedly. All the concurrent phenomena of menstruation are frequently present at the proper periods; but instead of the uterine discharge, there results great suffering to the patient by reason of the congestion of the pelvic organs or a vicarious discharge from the bladder, bowel or more distant points.

The first symptoms of superinvolution which attracts the attention of the patient is usually the continued suppression or very scanty discharge of the menstrual fluid. During lactation the menses are usually absent, and the patient first notices the abnormity soon after weaning the child. One, two, or three months may pass, and she becomes anxious, or fancies herself again pregnant; but as the months pass, and no other symptom of a new pregnancy appears, but on the contrary headache, backache, and a feeling of unrelieved distress in the pelvic organs occcurring periodically, she is at last made aware that something is wrong with her womb. Sometimes the breasts shrink, the subcutaneous adipose tissue begins to be absorbed, and the skin wrinkles, while the whole system is affected by the change in the uterus as it is at the climateric period when the uterus normally ceases its functional activity. Some patients are anæmic from the depression of the vital powers; others may be plethoric from the accumulation in the unrelieved circulatory system. Such was the condition in case IV.

But the distinctive characters of this condition of the uterus are found only by adding physical exploration to the rational symptoms already obtained. The abdomen may be normally full, but no uterine fundus can be felt by the most careful external palpitation. The vagina may be normal, but high up in its roof we feel a small nipple-shaped cervix, with a minute depression corresponding to the external os, or a mere depression where the cervix should be, and nothing corresponding to an os externum. The speculum shows the tissue pale. The sound, or perhaps only the pocket-probe, enters the uterine cavity two inches and a half, two inches, or barely an inch and a half. The organ is small, mobile, and when pushed with the sound against the rectum or abdominal wall, its own parietes are found to be exceedingly thin—so thin and friable that the sound has been accidentally pushed through into the peritoneal cavity. In the normal condition of the reproductive organs an ovary may sometimes be felt; but in the superinvolved condition of the uterus the ovaries, as shown by post-mortem examination, may become similarly affected and shrink to a comparatively small size, and the Graafian vessels disappear. In fact, the uterus may return to the ante-puberal size and become similar to the undeveloped uterus, as noticed in the first class of cases in this report. This will explain the symptoms, the headache, the general debility, the periodic congestions of the neighboring organs, and the sterility.

In the treatment of a superinvolved uterus regard must be had to the age of the patient, for if the climacteric period is near, little can be accomplished, and little is desired further than to relieve the immediate distress. But if the patient be young and in otherwise good health, the prognosis is by no means despairing. There, as in the case of congenitally deficient uterus, is the same double indication; once establish those two conditions, and the sterility will likely enough disappear. The peculiar infra-mammary pain in Case V. will be noticed hereafter.

Various means are proposed to increase the nutrition and restore the functions of the superinvolved uterus. The whole list of emmenagogues has been gone through over and over again, each in its day popular, and each failing to give satisfaction.

The Japanese and the Greeks of the time of Hippocrates are reported to have possessed specifics; but the one is too far in the past, and the other too far in the improbable, to fulfil our hopes and wishes. The only reliable means of remedy seems to be the uterine sound or pessary. So this condition of the uterus is similar to the condition already mentioned in the three first cases, as the indications and advantages are similar; and as I have there spoken sufficiently of the sound, it is proper to consider a means of cure which certainly surpasses all others.

The ordinary intra-uterine pessary is but a sound used continually instead of occasionally.

The galvanic pessary combines the advantages of the ordinary intra-uterine pessary with the powerful stimulus of the galvanic current.—*New York Medical Times for July*, 1864.

